# Influence of Social Determinants of Health on Presenting Visual Acuity in Retinal Vein Occlusions

**DOI:** 10.1016/j.xops.2026.101089

**Published:** 2026-01-29

**Authors:** Sidra Zafar, Bita Momenaei, Roselind Ni, Turner Wibbelsman, Martin Calotti, Theodore Bowe, Luis Acaba-Berrocal, Brian Cheng, Eric Kim, Na'il Hanif, Zafar Iqbal, Jordan D. Deaner, David Xu, Ajay E. Kuriyan, Yoshihiro Yonekawa, Jason Hsu, Sunir J. Garg, Julia A. Haller, Allen C. Ho, Samir N. Patel

**Affiliations:** 1 Wills Eye Hospital Retina Service, Mid Atlantic Retina, Thomas Jefferson University, Philadelphia, Pennsylvania; 2 University of Illinois, Urbana-Champaign, Illinois; 3 Department of Ophthalmology, Al-Shifa Hospital, Karachi, Pakistan; 4 The Vickie and Jack Farber Vision Research Center, Thomas Jefferson University, Philadelphia, Pennsylvania

**Keywords:** Retinal vein occlusion, Social determinants of health, Social deprivation index, Disparities

## Abstract

**Objective:**

To describe the impact of social determinants of health (SDOH) on visual acuity (VA) at presentation among patients with retinal vein occlusion (RVO).

**Design:**

A retrospective single-center cohort study.

**Subjects:**

All patients with RVO (central and branch retinal vein occlusion [CRVO and BRVO]) who received their first intravitreal anti-VEGF injection between January 2015 and March 2025 at a single center.

**Methods:**

Sociodemographic factors including age, gender, race, and ethnicity were recorded. Social deprivation index (SDI) scores, a proxy for SDOH were determined using zip codes. An aggregate SDOH score was calculated and divided into quintiles, with quintile 5 (Q5) representing the highest SDOH burden. Information on hypertension and smoking status were also collected. Data on best available VA at presentation measurements were collected and were categorized as mild (20/40 or better), moderate (20/50 to 20/100), and severe vision loss (20/200 or worse).

**Main Outcome Measures:**

Factors affecting degree of vision loss at presentation.

**Results:**

A total of 9698 patients with RVO were included. Of these, 57% (n = 5490/9698) had a BRVO, and 43% (n = 4208/9698) had a CRVO. The proportion of patients with mild, moderate, and severe vision loss was 25% (n = 2425/9698), 41.8% (n = 4049/9698), and 33.1% (n = 3206/9698), respectively. On multivariable analysis, increasing SDI quintiles (higher SDOH burden) were associated with worse presenting baseline VA in patients with RVO (odds ratio [OR] 1.32, 95% confidence interval [CI]: 1.13–1.54; *P* < 0.001) for patients in the fifth quintile versus those in the first quintile. Odds of severe vision loss at initial RVO presentation were also greater among older patients (OR 1.03, 95% CI: 1.02–1.03; *P* < 0.001), Hispanics (OR 1.62, 95% CI: 1.22–2.14; *P* = 0.001) versus non-Hispanics, and Black (OR 1.34, 95% CI: 1.17–1.34; *P* < 0.001) and Asian (OR 1.30, 95% CI: 1.02–1.67; *P* = 0.04) patients compared to White patients. Former smokers (OR 1.41, 95% CI: 1.17–1.70; *P* < 0.001) and never smokers (OR 1.39, 95% CI: 1.17–1.66; *P* < 0.001) were more likely to have mild vision loss at initial RVO presentation compared to active smokers.

**Conclusions:**

Our study reveals associations between adverse SDOH and presenting VA among patients with RVO. Evaluating and intervening on effects of adverse SDOH may be a means to improve outcomes of patients with RVO.

**Financial Disclosure(s):**

Proprietary or commercial disclosure may be found in the Footnotes and Disclosures at the end of this article.

Retinal vein occlusions (RVOs) are a relatively common cause of ocular morbidity and vision loss, with a 15-year cumulative incidence of 1.8% for branch retinal vein occlusions (BRVOs) and 0.5% for central retinal vein occlusions (CRVOs).^1^ Intravitreal anti-VEGF therapy is currently the main treatment for RVO with macular edema and its associated complications.^2^^,^^3^ Although anti-VEGF treatment is effective, presenting visual acuity (VA) remains one of the most important predictors for final visual outcome in RVO, independent of the drug used or the treatment regimen followed.^4^^,^^5^ However, little is known about sociodemographic factors that may influence presenting VA in patients with RVOs.

In a recent IRIS Registry (Intelligent Research in Sight) analysis,^6^ older patients and racial or ethnic minorities presenting with RVOs had worse baseline VA. There were also disparities noted in treatment initiation within 1 year of presentation, with older patients noted to be more likely to receive treatment and racial or ethnic minorities, and female patients were least likely to receive treatment.^6^ However, sociodemographic factors other than age, gender, race, and ethnicity also influence various aspects of health care, including access to and delivery of health care, severity of disease presentation, and outcomes. These social determinants of health (SDOH) can broadly be categorized into economic stability, education access and quality, health care access and quality, neighborhood and built environment, and social and community context.^7^ To optimize visual outcomes in patients from diverse backgrounds, this study seeks to describe the impact of SDOH on VA at presentation among patients with RVOs.

## Methods

This retrospective, single-center, cohort study received institutional review board approval from Wills Eye Hospital and adhered to the tenets of the Declaration of Helsinki. Informed consent was waived by the institutional review board.

### Data Collection

We included the initial presenting eye of all patients with an RVO (both CRVO and BRVO) who received their first intravitreal anti-VEGF injection between January 1st, 2015 and March 4th, 2025 and were evaluated at The Retina Service of Wills Eye Hospital and the offices of Mid Atlantic Retina. For patients with bilateral disease at presentation (n = 99 patients), the eye with worse VA was included. Baseline sociodemographic patient characteristics were collected by chart review. Race and ethnicity were self-reported by patients. The social deprivation index (SDI) analysis was performed using the 2019 SDI at the Zip Code Level dataset described previously.^8^ The SDI scores range from 1 to 100 and are a composite measure of area-level deprivation based on 7 demographic characteristics collected from the American Community Survey. The characteristics include the percentage living in poverty, percentage with less than 12 years of education, percentage of single-parent households, percentage living in a rented housing unit, percentage living in an overcrowded housing unit, percentage of households without a car, and percentage of unemployed adults younger than 65 years. Patient charts were also reviewed to collect information on the history of hypertension (HTN) and smoking status.

### Statistical Analysis

Data on the best available VA measurements were collected and were categorized as mild (20/40 or better), moderate (20/50–20/100), and severe vision loss (20/200 or worse). Sociodemographic and clinical characteristics (age, gender, race, ethnicity, SDI, HTN, and smoking status) were further assessed for a potential independent effect on (1) mild VA loss and (2) severe loss at presentation using univariable and multivariable linear regression models. Variables that resulted in a *P* value ≤ 0.2 from the univariable analyses were further evaluated using a multivariable linear regression model to adjust for confounding factors. All data were analyzed using STATA version 16. A 2-sided *P* value of < 0.05 was considered statistically significant.

## Results

### Baseline Demographics

A total of 9698 patients with RVO were included in our analysis. Of these, 57% (n = 5490/9698) had a BRVO and 43% (n = 4208/9698) had a CRVO. The mean (± standard deviation) patient age was 73.3 (±12.5) years. Most patients were White (82.6%, n = 7988/9698), and 2.5% were Hispanic (n = 242/9698) (Table 1).

**Table 1:** Baseline Characteristics of Patients Presenting with an RVO

	All RVO (N = 9698)	CRVO (n = 4208)	BRVO (n = 5490)
Mean age (standard deviation), yrs	73.3 (12.5)	73.7 (12.9)	73.3 (12.5)
Gender, No. (%)			
Female	4487 (46.3)	2104 (50)	2383 (43.4)
Male	5211 (53.7)	2104 (50)	3107 (56.6)
Race, No. (%)			
White	7988 (82.6)	3497 (83.1)	4491 (82.2)
Black	1251 (12.9)	523 (12.4)	728 (13.3)
Asian	303 (3.1)	123 (2.9)	180 (3.3)
Native American	35 (0.4)	15 (0.4)	20 (0.4)
Other	95 (1.0)	50 (1.2)	45 (0.8)
Ethnicity, No. (%)			
Non-Hispanic	9244 (95.6)	3910 (92.9)	5334 (97.6)
Hispanic	242 (2.5)	112 (2.7)	130 (2.4)
Other/declined	186 (1.9)	186 (4.4)	0 (0)
Social deprivation index, No. (%)			
1st quintile (1–20)	3842 (39.8)	1617 (38.6)	2225 (40.5)
2nd quintile (21–40)	2274 (23.6)	1023 (24.4)	1251 (22.8)
3rd quintile (41–60)	1597 (16.6)	707 (16.9)	890 (16.2)
4th quintile (61–80)	870 (9.0)	373 (8.9)	497 (9.1)
5th quintile (81–100)	1065 (11.0)	465 (11.1)	600 (10.9)
Smoking history, No. (%)			
Active	858 (8.9)	374 (8.9)	484 (8.9)
Former	2826 (29.3)	1268 (30.1)	1558 (28.6)
Never	5925 (61.4)	2520 (59.9)	3405 (62.5)
Unknown	46 (1.1)	46 (1.1)	0 (0)
Hypertension, No. (%)			
Yes	6087 (62.8)	2520 (59.9)	3567 (64.9)

BRVO = branch retinal vein occlusion; CRVO = central retinal vein occlusion; RVO = retinal vein occlusion.

### Baseline VA

In the BRVO cohort, the proportion of patients presenting with mild, moderate, and severe loss was 31.8% (n = 1748/5490), 41.2% (n = 2262/5490), and 27% (n = 1480/5490), respectively.

Among patients with CRVO, approximately 16% (n = 677/4208) had mild vision loss, 42.9% (n = 1806/4208) had moderate vision loss, and 41% (n = 1726/4208) had severe vision loss.

### Factors Associated with Mild Vision Loss

Tables 2 and 3 show the univariable and the multivariable analysis of factors associated with mild vision loss. In a multivariable analysis, increasing quintiles of SDI (increasing SDOH burden) were associated with a lower likelihood of patients presenting with mild vision loss. This association was seen even after adjusting for age, race, ethnicity, HTN, and smoking status. Older patients (odds ratio [OR] 0.97, 95% confidence interval [CI]: 0.97–0.97; *P* < 0.001), Black patients (OR 0.76, 95% CI: 0.65–0.88; *P* < 0.001), Asian patients (OR 0.71, 95% CI: 0.53–0.94; *P* = 0.02), and Hispanic patients (OR 0.72, 95% CI: 0.50–1.00; *P* = 0.04) were also less likely to present with mild vision loss compared to younger, White, and non-Hispanic patients.

**Table 2:** Univariable Analysis of Factors Associated with Mild Vision Loss at Presentation in Patients with RVOs

	All RVOs			CRVO			BRVO		
	Odds Ratio	95% CI	*P* Value	Odds Ratio	95% CI	*P* Value	Odds Ratio	95% CI	*P* Value
Age	0.98	0.97–0.98	<0.001	0.97	0.96–0.97	<0.001	0.98	0.97–0.98	<0.001
Gender (reference female)									
Male	1.02	0.93–1.12	0.68	1.17	0.99–1.38	0.06	1.06	0.94–1.19	0.34
Race (reference White)									
Black	0.78	0.68–0.91	0.001	0.89	0.69–1.14	0.35	0.72	0.60–0.86	<0.001
Asian	0.85	0.64–1.12	0.25	0.70	0.41–1.21	0.20	0.87	0.63–1.21	0.42
Native American	1.56	0.77–3.16	0.22	1.26	0.35–4.48	0.72	1.67	0.69–4.04	0.25
Other	0.63	0.36–1.11	0.11	0.56	0.22–1.42	0.22	0.74	0.38–1.44	0.38
Ethnicity (reference non-Hispanic)									
Hispanic	0.80	0.58–1.09	0.16	0.80	0.46–1.38	0.42	0.88	0.60–1.29	0.52
Social deprivation index (reference 1st quintile)									
2nd quintile (21–40)	0.91	0.81–1.03	0.14	1.05	0.85–1.29	0.67	0.88	0.76–1.02	0.10
3rd quintile (41–60)	0.84	0.74–0.97	0.02	0.86	0.67–1.10	0.22	0.86	0.73–1.02	0.08
4th quintile (61–80)	0.79	0.66–0.94	0.01	0.73	0.53–1.02	0.07	0.81	0.65–1.00	0.05
5th quintile (81–100)	0.74	0.63–0.88	<0.001	0.65	0.47–0.87	0.01	0.79	0.65–0.97	0.02
Smoking (reference active)									
Former	1.09	0.91–1.31	0.35	1.25	0.88–1.75	0.21	1.04	0.84–1.30	0.70
Never	1.14	0.96–1.35	0.13	1.27	0.92–1.76	0.14	1.07	0.87–1.32	0.50
Hypertension (reference no)									
Yes	1.09	0.99–1.20	0.10	1.01	0.85–1.20	0.94	1.05	0.93–1.18	0.42

BRVO = branch retinal vein occlusion; CI = confidence interval; CRVO = central retinal vein occlusion; RVO = retinal vein occlusion.

**Table 3:** Multivariable Analysis of Factors Associated with Mild Vision Loss at Presentation in Patients with RVOs

	All RVOs			CRVO			BRVO		
	Odds Ratio	95% CI	*P* Value	Odds Ratio	95% CI	*P* Value	Odds Ratio	95% CI	*P* Value
Age	0.97	0.97–0.97	<0.001	0.97	0.96–0.97	<0.001	0.98	0.97–0.98	<0.001
Gender (reference female)									
Male				0.99	0.84–1.17	0.92			
Race (reference White)									
Black	0.76	0.65–0.88	<0.001	0.89	0.67–1.17	0.39	0.69	0.57–0.84	<0.001
Asian	0.71	0.53–0.94	0.02	0.55	0.31–0.97	0.04	0.77	0.56–1.09	0.13
Native American	1.34	0.65–2.75	0.43	1.02	0.27–3.81	0.98	1.54	0.63–3.78	0.34
Other	0.69	0.40–1.21	0.20	0.54	0.21–1.39	0.20	0.72	0.36–1.47	0.37
Ethnicity (reference non-Hispanic)									
Hispanic	0.72	0.52–1.00	0.04				0.76	0.50–1.15	0.19
Social deprivation index (reference 1st quintile)									
2nd quintile (21–40)	0.91	0.81–1.03	0.14	1.01	0.81–1.24	0.96	0.88	0.76–1.03	0.11
3rd quintile (41–60)	0.83	0.72–0.95	0.002	0.78	0.60–1.00	0.05	0.87	0.73–1.03	0.11
4th quintile (61–80)	0.75	0.63–0.90	0.002	0.70	0.50–0.98	0.04	0.79	0.64–0.99	0.04
5th quintile (81–100)	0.77	0.65–0.92	0.004	0.63	0.45–0.87	0.01	0.87	0.70–1.07	0.21
Smoking (reference active)									
Former	1.41	1.17–1.70	<0.001	1.72	1.21–2.43	0.002			
Never	1.39	1.17–1.66	<0.001	1.57	1.13–2.18	0.01			
Hypertension (reference no)									
Yes	1.14	1.03–1.25	0.01						

BRVO = branch retinal vein occlusion; CI = confidence interval; CRVO = central retinal vein occlusion; RVO = retinal vein occlusion.

Former smokers (OR 1.41, 95% CI: 1.17–1.70; *P* < 0.001) and never smokers (OR 1.39, 95% CI: 1.17–1.66; *P* < 0.001) were more likely to have mild vision loss at initial RVO presentation compared to active smokers. Patients with a known history of HTN (OR 1.14, 95% CI: 1.03–1.25; *P* = 0.01) were also more likely to present with mild vision loss at initial presentation with an RVO.

### Factors Associated with Severe Vision Loss

Tables 4 and 5 show the univariable and multivariable analysis of factors associated with severe vision loss. Patients in the fourth (OR 1.19, 95% CI: 1.01–1.39; *P* = 0.04) and fifth quintile of SDI (OR 1.32, 95% CI: 1.13–1.54; *P* < 0.001) (highest SDOH burden) had increased odds of presenting with severe vision loss compared to patients in the first quintile (least SDOH burden). Odds of severe vision loss at initial RVO presentation were also greater among older patients (OR 1.03, 95% CI: 1.02–1.03; *P* < 0.001) versus younger, Hispanic patients (OR 1.62, 95% CI: 1.22–2.14; *P* = 0.001) versus non-Hispanics and Black (OR 1.34, 95% CI: 1.17–1.53; *P* < 0.001) and Asian (OR 1.30, 95% CI: 1.02–1.67; *P* = 0.04) patients compared to White patients.

**Table 4:** Univariable Analysis of Factors Associated with Severe Vision Loss at Presentation in Patients with RVO

	All RVOs			*CRVO*			*BRVO*		
	Odds Ratio	95% CI	*P* Value	Odds Ratio	95% CI	*P* Value	Odds Ratio	95% CI	*P* Value
Age	1.02	1.02–1.03	<0.001	1.03	1.02–1.03	<0.001	1.02	1.02–1.03	<0.001
Gender (reference female)									
Male	0.92	0.85–1.01	0.08	0.86	0.76–0.97	0.02	0.91	0.80–1.02	0.11
Race (reference White)									
Black	1.30	1.14–1.47	<0.001	1.17	0.97–1.41	0.10	1.44	1.22–1.71	<0.001
Asian	1.13	0.89–1.45	0.31	0.98	0.67–1.43	0.90	1.31	0.95–1.81	0.10
Native American	1.02	0.49–2.09	0.97	1.10	0.38–3.17	0.86	0.97	0.35–2.66	0.95
Other	1.79	1.16–2.7	0.01	2.51	1.29–4.87	0.01	1.31	0.69–2.47	0.41
Ethnicity (reference non-Hispanic)									
Hispanic	1.49	1.15–1.94	0.003	1.49	1.02–2.17	0.04	1.40	0.97–2.03	0.07
Social deprivation index (reference 1st quintile)									
2nd quintile (21–40)	1.08	0.97–1.21	0.16	0.96	0.81–1.13	0.62	1.17	1.00–1.37	0.05
3rd quintile (41-60)	1.11	0.97–1.25	0.12	1.03	0.86–1.24	0.72	1.13	0.95–1.35	0.16
4th quintile (61–80)	1.16	0.99–1.35	0.07	1.19	0.95–1.50	0.14	1.13	0.91–1.41	0.27
5th quintile (81–100)	1.43	1.24–1.65	<0.001	1.37	1.11–1.70	0.003	1.46	1.20–1.78	<0.001
Smoking (reference active)									
Former	1.03	0.88–1.21	0.72	1.14	0.90–1.45	0.28	0.93	0.75–1.17	0.54
Never	0.96	0.82–1.11	0.56	1.12	0.89–1.40	0.33	0.85	0.68–1.04	0.12
Hypertension (reference no)									
Yes	1.01	0.93–1.11	0.80	1.14	1.01–1.30	0.04	0.97	0.86–1.10	0.67

BRVO = branch retinal vein occlusion; CI = confidence interval; CRVO = central retinal vein occlusion; RVO = retinal vein occlusion.

**Table 5:** Multivariable Analysis of Factors Associated with Severe Vision Loss at Presentation in Patients with RVO

	All RVOs			CRVO			BRVO		
	Odds Ratio	95% CI	*P* Value	Odds Ratio	95% CI	*P* Value	Odds Ratio	95% CI	*P* Value
Age	1.03	1.02–1.03	<0.001	1.03	1.03–1.04	<0.001	1.02	1.02–1.03	<0.001
Gender (reference female)									
Male	1.02	0.94–1.12	0.59	0.96	0.84–1.09	0.51	0.99	0.87–1.12	0.82
Race (reference White)									
Black	1.34	1.17–1.53	<0.001	1.23	1.01–1.51	0.04	1.49	1.24–1.79	<0.001
Asian	1.30	1.02–1.67	0.04	1.20	0.82–1.74	0.36	1.53	1.10–2.13	0.01
Native American	1.17	0.57–2.39	0.68	1.39	0.48–3.97	0.54	1.05	0.38–2.94	0.93
Other	1.54	1.00–2.39	0.05	1.87	1.02–3.41	0.04	1.10	0.55–2.19	0.79
Ethnicity (reference non-Hispanic)									
Hispanic	1.62	1.22–2.14	0.001	1.57	1.05–2.36	0.03	1.70	1.13–2.56	0.01
Social deprivation index (reference 1st quintile)									
2nd quintile (21–40)	1.09	0.98–1.22	0.13	0.99	0.84–1.17	0.94	1.15	0.98–1.35	0.08
3rd quintile (41–60)	1.10	0.97–1.26	0.12	1.09	0.91–1.31	0.35	1.08	0.90–1.30	0.39
4th quintile (61–80)	1.19	1.01–1.39	0.04	1.24	0.98–1.57	0.07	1.12	0.89–1.40	0.33
5th quintile (81–100)	1.32	1.13–1.54	<0.001	1.34	1.06–1.66	0.01	1.26	1.01–1.56	0.04
Smoking (reference active)									
Former							0.75	0.59–0.94	0.01
Never							0.70	0.56–0.87	0.001
Hypertension (reference no)									
Yes				1.01	0.88–1.15	0.91			

BRVO = branch retinal vein occlusion; CI = confidence interval; CRVO = central retinal vein occlusion; RVO = retinal vein occlusion.

## Discussion

In this retrospective analysis of nearly 10 000 patients over 10 years, adverse SDOH was associated with worse VA in patients with RVO at initial presentation. In addition, being a racial or ethnic minority and older age were associated with worse VA at initial presentation with an RVO.

Understanding the social needs of patients is essential for effective delivery of eye care, especially to the most vulnerable populations. Adverse SDOH can present barriers to timely presentation to physicians and limit vision outcomes. Places with concentrated poverty often lack health care services and have low levels of health care utilization.^9^ Indeed, socioeconomic disadvantage has been associated with underuse of health care and poor health outcomes across various eye diseases, including diabetic retinopathy (DR), age-related macular degeneration, rhegmatogenous retinal detachment, and glaucoma.^10^, ^11^, ^12^, ^13^ Patel et al found an increased prevalence of severe vision loss among patients presenting with exudative age-related macular degeneration as regional adjusted gross income decreased.^10^ Socioeconomic factors have also been shown to influence macula status at rhegmatogenous retinal detachment presentation.^11^ Lower educational attainment and lower income levels were associated with increased mortality risk following RVO in a population-based Danish study.^14^ Neighborhood socioeconomic disadvantage has also been associated with a greater risk of DR, including proliferative DR and lower rates of DR screening.^15^^,^^16^ In our study, patients from the most socioeconomically deprived neighborhoods were 32% more likely to have severe vision loss upon initial presentation (Table 5). Additionally, patients in the third, fourth, and fifth quintiles of SDI were 17%, 25%, and 23% less likely to have VA of 20/40 or better at presentation compared to those in the first quintile, respectively (Table 3). In addition to disparities in the US health care system, health behaviors such as use of tobacco, lack of exercise, and poor diet also vary by socioeconomic status.^17^ Less robust social support networks and less access to high-quality health care often characterize living conditions in socioeconomically disadvantaged neighborhoods.^17^^,^^18^

Racial and ethnic disparities affecting severity of disease presentation have been reported extensively across multiple studies.^6^^,^^19^, ^20^, ^21^ Our finding of Black, Asian, and Hispanic patients presenting with more severe vision loss at initial RVO presentation is similar to Haller et al.^6^ In their analysis of over 304 000 patients with RVO, Hispanic patients and non-White patients presented with worse VA and more severe disease than White and non-Hispanic patients. Black patients were also 10% less likely to receive anti-VEGF treatment in the first 12 months after initial RVO presentation compared to White patients.^6^ Similar findings were also reported by Kiew et al, who found presenting VA was worse among Black patients with CRVO compared to White patients.^19^ Possible reasons may include a lack of awareness, medical distrust, and lower rates of eye care utilization among these populations.^21^

Older age has been associated with poor visual prognosis in several CRVO studies.^4^^,^^22^ In a post hoc analysis of the LEAVO study, mean VA gains decreased by 0.33 for every year of older age.^4^ Similar findings were also reported by the investigators of the SCORE and SHORE studies.^23^^,^^24^ Additionally, in the study by Haller et al, approximately 21% of patients aged ≥81 years versus 12% to 15% among those aged ≤80 years had VA worse than 20/200.^6^ Although we did not specifically evaluate visual outcomes in our study, it is possible that more severe vision loss at presentation among older individuals may be one of the factors limiting final VA gain in this population. This is consistent with prior studies where presenting VA was among the most important predictors for final visual outcomes in RVOs.^4^^,^^5^

Smoking is a significant risk factor for various vascular diseases, including coronary artery disease, peripheral artery disease, and stroke.^25^^,^^26^ Several authors have assessed the association between smoking and RVOs with conflicting evidence.^27^, ^28^, ^29^ In the Beaver Dam Eye Study, current smoking quadrupled the incidence of BRVO.^28^ Similar results were reported by Lee et al who found an increased prevalence of BRVO with cigarette smoking in the Korean National Health and Nutrition Survey database.^27^ In contrast, no association was noted for smoking and RVO prevalence in the Beijing Eye Study.^29^ In our study, active smoking was associated with lower odds of presenting with mild vision loss, thereby identifying a potential modifiable risk factor for severity of disease presentation in RVO.

Finally, we found patients with a known history of HTN were more likely to have mild vision loss at presentation versus those without a known HTN history. No associations were noted between HTN history and severe vision loss. We hypothesize that known hypertensives may be more likely to be on anti-hypertensive medications and may have better disease control. However, more studies are needed to corroborate our findings.

There are several limitations of this study. Best available VA was obtained using current correction or pinhole instead of a standardized protocol refraction. It is possible that other ocular comorbidities could have influenced presenting VA, such as cataract, DR, or age-related macular degeneration; however, this study design may be more generalizable to clinical settings in which patients may have multiple active ocular conditions. Furthermore, the presenting VA of RVOs in this study is comparable to recent real-world registry studies evaluating RVO outcomes.^6^^,^^30^ Another limitation is that this study was unable to determine the time from symptom onset to presentation to the office. Patients with delayed presentations may be more likely to have a severe vision loss at the initial visit. These findings may be exacerbated by higher SDOH burden, which can also present barriers to timely presentation to treating physicians. Another limitation is that it is possible that patients with newly diagnosed RVO may have initially been followed by outside providers prior to their referral to our practice. Finally, the results of this single-center study may not be representative of patients with RVO in other practices or regions.

This large, retrospective study highlights that adverse SDOH, older age, racial and ethnic minority status, and active smoking were all independently associated with worse VA at initial presentation for RVO. These findings underscore the importance of recognizing how social and demographic factors influence ocular health and disease severity. Targeted public health strategies aimed at improving access to eye care and early intervention in socioeconomically disadvantaged and minority populations may help mitigate vision loss from RVO. Further research is needed to better understand the mechanisms underlying these disparities and to develop interventions that address both clinical and social contributors to disease burden.
